# Supplementary material for: Integrated Microbiota and Metabolome Analysis to Assess the Effects of the Solid-State Fermentation of Corn–Soybean Meal Feed Using Compound Strains
Source: Microorganisms. 2023 May 17;11(5):1319. doi: 10.3390/microorganisms11051319 (PMC10221456; doi:10.3390/microorganisms11051319)
Supplement: Supplementary file 1 [file microorganisms-11-01319-s001.zip › microorganisms-2361408-supplementary.pdf]

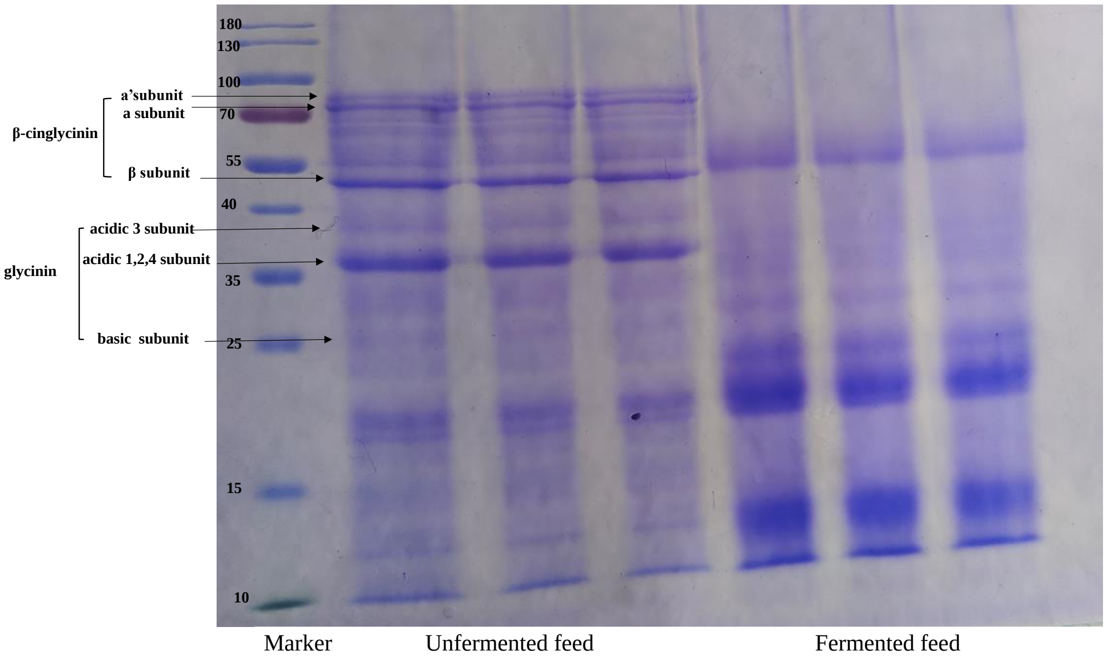

Figure S1. Distribution of peptides in unfermented and fermented feed.

Protein molecular weight marker (10–100 kDa) and unfermented and fermented feed samples
